# The DDX23 Negatively Regulates Translation and Replication of Foot-and-Mouth Disease Virus and Is Degraded by 3C Proteinase

**DOI:** 10.3390/v12121348

**Published:** 2020-11-25

**Authors:** Sahibzada Waheed Abdullah, Shichong Han, Jin’en Wu, Yun Zhang, Manyuan Bai, Ye Jin, Xiaoying Zhi, Junyong Guan, Shiqi Sun, Huichen Guo

**Affiliations:** State Key Laboratory of Veterinary Etiological Biology, O.I.E./China National Foot-and-Mouth Disease Reference Laboratory, Lanzhou Veterinary Research Institute, Chinese Academy of Agricultural Sciences, Lanzhou 730046, China; waheed_149@yahoo.com (S.W.A.); hanshichong081@126.com (S.H.); wujinen@caas.cn (J.W.); zhangyun03@caas.cn (Y.Z.); baimanyuan@caas.cn (M.B.); jinye@caas.cn (Y.J.); zhixiaoying333@163.com (X.Z.); guanzky@163.com (J.G.)

**Keywords:** foot-and-mouth disease virus, internal ribosome entry site, replication, 3C proteinase, DEAD-box helicase 23

## Abstract

DEAD-box helicase 23 (DDX23) is a host nuclear helicase, which is a part of the spliceosomal complex and involved in pre-mRNA splicing. To investigate whether DDX23, an internal ribosomal entry sites transacting factor (ITAF) affects foot-and-mouth disease virus (FMDV) replication and translation through internal ribosome entry site (IRES)-dependent manner. For this, we utilized a pull-down assay, Western blotting, quantitative real-time PCR, confocal microscopy, overexpression and small interfering RNA knockdown, as well as the median tissue culture infective dose. Our findings showed that FMDV infection inhibited DDX23 expression and the overexpression of DDX23 reduced viral replication, however, CRISPR Cas9 knockout/small interfering RNA knockdown increased FMDV replication. FMDV IRES domain III and IV interacted with DDX23, whereas DDX23 interacted with FMDV 3C proteinase and significantly degraded. The enzymatic activity of FMDV 3C proteinase degraded DDX23, whereas FMDV degraded DDX23 via the lysosomal pathway. Additionally, IRES-driven translation was suppressed in DDX23-overexpressing cells, and was enhanced in DDX23 knocked down. Collectively, our results demonstrated that DDX23 negatively affects FMDV IRES-dependent translation, which could be a useful target for the design of antiviral drugs.

## 1. Introduction

Foot-and-mouth disease (FMD) affects the health of cattle, goats, sheep, swine, and wild cloven-hoofed animals [1]. FMD halts the animal trade between FMD-free and prevalent countries, and could be used potentially as a bioweapon. North and South America, Western Europe, New Zealand and Australia are free of FMDV, whereas the remaining countries throughout the world are enzootic. The etiological agent, foot-and-mouth disease virus (FMDV), is a filterable, RNA plus-strand virus with icosahedral symmetry and a genome of 8500 bases. It belongs to the genus *Apthovirus* in the family *Picornaviridae* [2]. There are seven FMDV serotypes, O, A, C, Asia1, SAT I, SAT II, and SAT III [3,4,5]. The FMDV genome is composed of a 5′ untranslated region (5′UTR), a single long open reading frame (ORF), and 3′UTR [6]. These non-coding regions are of great importance for viral RNA stability, virulence, and replication [7].

The internal ribosome entry site (IRES) is a crucial sequence in RNA viruses. It is important in gene expression and the translation initiation of viral and eukaryotic cell mRNAs via the noncanonical pathway [8]. FMDV IRES belongs to type II IRES, which is 450 nucleotides long [9] and contains five domains (I–V). Domain I functions like a cis replication element (cre) [6], whereas domains II-IV are mainly involved in IRES activity [10]. Domain III of the FMDV IRES was found to be crucial for the attachment with IRES transacting factors and RNA–RNA interaction [11]. Domain V has an AUG motif which is recognized by host proteins, pyrimidine tract binding protein (PTB), and translation initiation factor 4B (EIF4B) [12,13].

The IRES of FMDV strongly dependent on cellular helicases and IRES transacting factors (ITAFs). ITAFs such as PCBP2, PTB, ERBB3-binding protein 1, FBP2, FBP1, hnRNPA1, G3BP1, and CSDE1, ARF5, Rab1b, Gemin5, Sam68, hnRNPK, [14,15,16,17,18,19,20,21,22] may have IRES-promoting or repressing activity [23]. For example, polypyrimidine tract-binding protein (PTB) is the component of the 80S and 48S ribosomal initiation complex formed when FMDV internal ribosome entry site RNA recruits the ribosome. At first, the PTB-IRES complex formation occurs, followed by joining the small ribosomal subunit and promoting the FMDV IRES translation [24]. Conversely, an ITAF, Gemin5, is the negative regulator of FMDV IRES activity. Pull-down assay revealed that Gemin5 forms two complexes, eIF4E containing IRES–independent complex and ribonucleoprotein–IRES complex, which play critical roles in translation regulatory activity [20]. Recently, it was identified that novel ITAF, heterogeneous nuclear ribonucleoprotein (hnRNPK), a negative regulator of FMDV IRES translation, interacted with the FMDV IRES II, III and IV domains via KH2 and KH3 domain sites and inhibited the viral replication [22]. To this end, the virus–host interplay is a crucial step in viral replication. Therefore, to understand the virus replication and for control strategies, the discovery of novel ITAFs warrants further research.

The previous studies demonstrated that DEAH-box and DEAD-box proteins, which belong to the RNA helicase family, are considered vital pillars of viral replication and have been associated with biological activities like transcription, metabolism, RNA splicing, and translation [25,26]. It was suggested that the RNA helicases played pivotal roles during virus–host association, and have been found to upregulate the IRES functions or act as cellular sensors to defend cells from invading viruses [27]. The DEAD-box helicase 23 (DDX23) is a helicase belonging to the DEAD-box proteins family. It is a core component of the U5 small nuclear ribonucleoproteins snRNP, involved in RNA splicing [28,29]. The knockdown of DDX23 significantly decreased the interferon-stimulated genes secretion from the vesicular stomatitis virus (VSV) infected cells, and was involved in binding with poly(I:C) and sensing of RNA viruses [30]. Furthermore, DDX23 was involved in various infectious processes of Dengue virus, human immunodeficiency virus (HIV), chronic Hepatitis B virus, and Astrovirus [28,31,32,33]. However, viruses have evolved various strategies to efficiently cleave or modify these cellular ITAFs to replicate in the host cells [34].

FMDV 3C proteinase (3C^pro^) is one of the highly conserved genes within the viral genome responsible for the cleavage of ten out of thirteen FMDV polyproteins [34,35]. Moreover, FMDV 3C proteinase was involved in host-virus interactions and cleavage of various host proteins [36]. It was suggested that the FMDV 3C^pro^ utilizes catalytic tools, such as D84, C163, and H46Y, for the cleavage of FMDV polyproteins and various host cellular proteins [37,38,39,40,41]. During the onset of viral infection, the type 1 interferon (*IFN1*) and cytokines are promptly induced against the viral infection [42], whereas 3C^pro^ is involved in the inhibition of IFN-α/β by blocking the nuclear translocation of STAT1/STAT2 [1]. Hence, FMDV 3C^pro^ is considered a potential target for antiviral drugs [43].

In the current study, we showed that DDX23 directly binds to the FDMV IRES. Overexpression of DDX23 decreases the FMDV translation and replication. Furthermore, the waning of DDX23 protein was observed with FMDV infection compared to that of the uninfected cells. The viral protein 3C^pro^ interacted with DDX23 was held accountable for the decrease in DDX23. Taken together, our results demonstrated that DDX23 has a novel function to restrict the FMDV infection, which will help for the development of new strategies against viral infection and the production of potent novel vaccines in near future.

## 2. Materials and Methods

### 2.1. Cells and Virus

PK-15 cells (porcine kidney, ATCC^®^ CCL33™) were cultured in Dulbecco’s modified Eagle’s medium (Gibco Laboratories, Carlsbad, CA, USA) supplemented with 10% fetal bovine serum (Gibco, Carlsbad, CA, USA), and penicillin–streptomycin (100 U mL^−1^ and 100 g mL^−1^, respectively) (Gibco) at 37 °C under 5% CO_2_.

The FMDV serotype O strain O/BY/CHA/2010 (GenBank accession no. JN998085.1) was provided by the O.I.E./National Foot-and-mouth Disease Reference Laboratory of China (Lanzhou, China). FMDV was propagated in BHK-21 cells (baby hamster kidney, ATCC^®^ CCL-10™). The viral titers were determined as the 50% tissue culture infective dose (TCID_50_) in BHK-21 cells.

### 2.2. Antibodies and Reagents

Anti-DDX23, DDX3 antibodies were purchased from Abcam (Cambridge, MA, USA). The Anti-FLAG antibody was purchased from Proteintech (Chicago, IL, USA). Anti-hemagglutinin (HA) antibody, secondary antibodies conjugated with either horseradish peroxidase (HRP) or fluorescein isothiocyanate (FITC), and the chloroquine phosphate inhibitor was purchased from Sigma-Aldrich (St. Louis, MO, USA). The anti-actin antibody was purchased from Santa Cruz Biotechnology (CA, USA). The inhibitor Z-VAD(OMe)-FMK was purchased from Cell Signaling Technology (Danvers, MA, USA). XhoI and proteinase K were purchased from New England Biolabs (NEB, Ipswich, MA) Polyclonal pig antiserum directed against FMDV was prepared in our laboratory. MG-132 was purchased from Selleck Chemicals (Houston, TX, USA).

### 2.3. Biotinylated RNA Pull-Down Assay

The full-length 5′UTR, 3′UTR, and IRES of FMDV were synthesized with the T7 RiboMAX™ Express Large-Scale RNA Production System (Promega) (Madison, WI, USA). The RNA-protein pull-down assay was performed with the Pierce™ Magnetic RNA–Protein Pull-Down Kit (Thermo Scientific Pierce, Rockford, IL, USA). Briefly, 50 pmol of 5′UTR, 3′UTR, IRES, IRES d1-2, d3-5, d3, d4-5, d4, or d5 mRNAs were labeled with desthiobiotin using T4 RNA ligase, with the Pierce™ RNA 3′ End Desthiobiotinylation Kit (Thermo Scientific Pierce, Rockford, IL, USA), and incubated individually with prewashed streptavidin magnetic beads (50 µL) in RNA capture buffer for 15–30 min at room temperature with agitation to allow binding. Unlabeled 5′UTR, 3′UTR, IRES, IRES d1-2, d3-5, d3, d4-5, d4, or d5 mRNA was used as the control. The RNA-bound beads were then equilibrated in Protein-RNA Binding Buffer (Thermo Scientific Pierce), and 40 µg of PK-15 cell lysate proteins were added. Each sample was incubated for 60 min at 4 °C with rotation. The beads were then washed with wash buffer, vortexed, and separated on a magnetic stand. The samples were eluted with 50 µL of nondenaturing Biotin Elution Buffer (Thermo Scientific Pierce) and assessed with silver staining, mass spectrometry, and Western blotting.

### 2.4. Plasmid Construction and Transfection

The full-length coding region of DDX23 was amplified from the total RNA of PK-15 cells with RT-PCR using primers 5′-CGGGATCCATGGCAGGAGAGCTGGCTGATA-3′ and 5′-GGAATTCTCAGGCAAAGATGGTCTCTTCCC-3′, containing BamHI (TaKaRa, Dalian, China) and EcoRI (TaKaRa) restrictions sites, respectively, which were designed according to the DDX23 mRNA sequence (accession no. XM_021091587). Briefly, reverse transcription (RT) was performed with the reverse transcriptase master mix (TaKaRa). PCR amplification was performed with PrimeSTAR GXL DNA Polymerase (TaKaRa), and the PCR products were purified with the E.Z.N.A^®^ Gel Extraction Kit (Omega, Norcross, GA, USA), according to the manufacturers’ protocols. The purified DNA fragment was inserted into the BamHI and EcoRI sites in the pCMV-N-Flag vector (Beyotime Biotechnology, Shanghai, China) and expressed as the tagged recombinant protein Flag–DDX23 (NCBI accession no. MT815983). The sequence of the insert was confirmed with the restriction enzyme analysis and DNA sequencing. Plasmids expressing FMDV proteins 2B, 2C, 3A, 3C, 3D, L, VP0, VP1, or VP3 were designated, p2B-flag, p2C-flag, p3A-flag, p3C-flag, p3Dflag, pL-flag, pVP0-flag, pVP1-flag, pVP3-flag, p3C-H46Y-flag, p3C-D84N-flag, p3C-163G-flag, or p3C-H205R, respectively, and stored in our laboratory [44]. According to the manufacturer′s protocol, transfection was performed with Lipofectamine^®^ LTX Reagent with Plus Reagent (Invitrogen, Carlsbad, CA, USA). Bicistronic reporter plasmids were constructed as previously described [45]. Briefly, the FMDV IRES sequence (IRES plus 69 bases of the coding region) fragment was cloned into the psiCHECK-2 vector (Promega, Madison, WI, USA). All DNA constructs were verified with DNA sequencing [46].

### 2.5. In vitro Transcription

The T7 promoter-FMDV IRES plasmids were linearized with XhoI and purified by phenol-chloroform extraction. RNA transcripts were synthesized using a RiboMAX^TM^ Large Scale RNA Production System-T7 kit (Promega, Madison, WI, USA) according to the manufacturer’s protocol. Biotinylated RNA was synthesized by using Pierce^TM^ RNA 3′ End Desthiobiotinylation kit (Promega, Madison, WI, USA) according to the manufacturer′s protocol.

### 2.6. Knockdown and FMDV Infection

PK-15 cells were transfected with small interfering (si)RNA targeting the DDX23 mRNA of *Sus scrofa*. The *Sus scrofa* siRNA sequences were 5′-GGAAACAGUUCCAAGACUUTT-3′ and 5′-AAGUCCUUGGAACUGUUUCCTT-3′, and the negative control (NC) siRNA sequences were 5′-UUCUCCGAACGUGUCACGUTT-3′ and 5′-ACGUGACACGUUCGGAGAATT-3′. All siRNAs were prepared commercially by GenePharma (Shanghai, China). According to the manufacturer′s instructions, the DDX23-containing plasmid was delivered into the cells with Lipofectamine RNAiMAX (Invitrogen). After 35 h, the transfected cells were infected with FMDV type O at a multiplicity of infection (MOI) of 0.5. Samples were collected after 0, 3, 6, and 9 h and used for downstream applications.

### 2.7. Western Blotting

The transfected and infected PK-15 cells were lysed with 1 X SDS buffer. An equal amount of each sample was separated with 10% SDS-PAGE and transferred to nitrocellulose membrane. Polyclonal pig antiserum against FMDV was prepared by our laboratory (diluted 1:1000), followed by an HRP-conjugated anti-pig IgG secondary antibody (1:1000). DDX23 was detected in the siRNA-treated (knockdown) and pCMV-N-Flag–DDX23-transfected cells with anti-Flag antibodies. β-Actin was detected on the same membrane with a mouse anti-β-actin antibody, as the loading control.

### 2.8. TCID_50_ Assay

The supernatants of PK-15 cells were collected, centrifuged, and diluted 10-fold. Supernatant samples (100 μL) and viral stock were added to BHK-21 cells, which were then seeded in a 96-well plate at 90% confluence for about 72 h at 37 °C. The cytopathic effects in the wells were counted. The TCID_50_/100 μL values were calculated with the Reed-Muench method. Each test was performed at least three times.

### 2.9. RNA Extraction and qRT–PCR Analysis

The total RNA was extracted from PK-15 cells with TRIzol Reagent (Invitrogen) as previously described [47]. The concentration and purity (measured as the absorbance ratios A_260/280_ and A_260/230_, respectively) of the isolated RNA were determined with a NanoDrop spectrophotometer (Thermo Scientific, Wilmington, DE, USA). The RT reaction was performed with 1 µg of total RNA and PrimeScript 5× RT Master Mix (TaKaRa). The cDNA was stored at −20 °C until further analysis. According to the manufacturer′s instructions, qPCR was performed with SYBR^®^ Premix Ex Taq II (Tli RNaseH Plus) (TaKaRa). Briefly, the 20 µL reaction mixture contained 10 µL of SYBR^®^ Premix Ex Taq II, 0.8 µL (16 µM) of each primer (forward and reverse), 0.4 µL of Rox reference dye II (50×), 2 µL of cDNA, and 6 µL of RNase-free water. Glyceraldehyde 3-phosphate dehydrogenase (GAPDH)-directed primers were used as the endogenous reference. The primers specific for the GAPDH were 5¢- ACATGGCCTCCAAGGAGTAAGA-3′ (sense) and 5′- GATCGAGTTGGGGCTGTGACT-3′ (antisense). The primers specific for the FMDV were 5′-CAAACCTGTGATGGCTTCGA-3′ (sense) and 5′-CCGGTACTCGTCAGGTCCA-3′ (antisense). The primers specific for the DDX23 were 5′-ATGGAAATGAGGACGAGGAGG- 3′ (sense) and 5′- AACTTGCGGTCGTTGAGATG-3′ (antisense). Forty qPCR cycles were performed with the following cycling conditions: 15 s at 95 °C and 30 s at 60 °C. All reactions were performed in triplicate and were analyzed with the ABI PRISM 7300 Sequence Detection System (Applied Biosystems, Foster, CA, USA).

### 2.10. Confocal Microscopy

The PK-15 cells seeded on the bottoms glass cell culture dishes (NEST, Jiang Xi, China) were transfected with the indicated plasmid. After 30 h, the cells were infected with FMDV type O (MOI = 5), and the samples were collected at 0, 3, and 6 h post-infection (hpi). The cells were fixed with 4% paraformaldehyde for 10 min at room temperature and then permeabilized for 10 min with phosphate-buffered saline (PBS) containing 0.1% Triton X-100 and 5% newborn calf serum (NBS) (Gibco, Carlsbad, CA, USA), and blocked with 5% NBS for 1 h at 37 °C in an incubator. The cells were then incubated with a mouse monoclonal anti-Flag primary antibody (diluted 1:100). FMDV was stained with Polyclonal pig antiserum against FMDV prepared in our laboratory (diluted 1:100) overnight at 4 °C. After the cells were washed five times with PBS (6 min each wash), they were incubated for 1 h at 37 °C in a humid incubator with fluorochrome-conjugated secondary antibodies. Finally, the nuclear DNA was stained with 4′,6-diamidino-2-phenylindole (Beyotime Biotechnology, Shanghai, China) for 20 min at room temperature. All the specimens were analyzed with a laser-scanning confocal microscope (Leica SP8, Leica Micro-system, Solms, Germany) at wavelengths of 405, 488, and 561 nm.

### 2.11. Luciferase Reporter Assay

Cells were transfected with polypyrimidine tract-binding protein 1 (PTBP1), DDX23-directed siRNA or pCMV-N-FLAG-DDX23 or pCMV-N-FLAG-DDX3 plasmid, with the indicated bicistronic constructs. The luciferase assay was performed with the Dual-Luciferase^®^ Reporter Assay System (Promega, Madison, WI, USA) 24 h after transfection. At 24 h post-transfection, cell extracts were generated with a passive buffer and examined for RLuc and FLuc activity with the Dual-Glo^®^ Luciferase Assay System (Promega, Madison, WI, USA).

### 2.12. Immunoprecipitation Assay

PK-15 cells were lysed with radioimmunoprecipitation assay RIPA lysis buffer (Beyotime Biotechnology) for 1 h on ice and then centrifuged at 15,000× *g* for 20 min at 4 °C. The supernatants were immunoprecipitated with gentle rotation using the appropriate antibodies at 4 °C overnight. The immune complexes were incubated with Protein G Sepharose 4 Fast Flow (GE Healthcare Bio-Sciences AB, Uppsala, Sweden) for 2 h at 4 °C, washed four times with lysis buffer, and finally eluted with 1 × SDS-PAGE sample buffer for Western blotting analysis.

### 2.13. RNA Immunoprecipitation and RT–PCR

The lysates of the FMDV-infected PK-15 cells used in the immunoprecipitation assays were collected at 5 hpi and preincubated with Protein G Sepharose 4 Fast Flow (GE Healthcare Bio-Sciences AB, Uppsala, Sweden) on ice for 1 h. Nonspecific complexes were pelleted by centrifugation at 1000× *g* for 10 min at 4 °C. The supernatants were recovered, and 100 µL samples were diluted with 500 µL of lysis buffer. The indicated antibodies (4 µL) were then added and the samples incubated on ice for 2 h. Prewashed Protein G Sepharose 4 Fast Flow (50 µL) was added to each sample, then incubated on ice for 1 h. The RNA-protein coimmunoprecipitation complexes were pelleted by centrifugation at 1000× *g* for 5 min at 4 °C and washed three times with lysis buffer. Each pellet was resuspended in 400 µL of proteinase K buffer (100 mM Tris-HCl [pH 8.0], 12.5 mM EDTA, 150 mM NaCl, 1% SDS) and incubated with 100 µg of predigested proteinase K (NEB) for 30 min at 37 °C. The RNA was extracted with TRIzol Reagent (Invitrogen) and amplified by RT-PCR. RT-PCR was performed with the PrimeScript One-Step RT-PCR Kit (TaKaRa) and primers specific for the FMDV IRES (5′-CACAGGTTCCCACAACCGACAC-3′ and 5′-CAGTGATAGTTAAGGAAAGGC-3′); specific for the 3′UTR (5′-GTTGCTAGTGATTATGACTTGGAC-3′ and 5′-CTTACGGCGTCGCTCGCCTCAGAG-3′); specific for RPS16 RNA (5′-TCGCAGCCATGCCGTCCAAGGGT-3′ and 5′-TCATTAAGATGGGCTCATCGGT-3′); or specific for *GAPDH* RNA (5′-TCCATGCCATCACGGCCACCCAG-3′ and 5′-ACTCTTGAAGTCGCAGGAGACAAC-3′) were used. The PCR products were resolved in 1% agarose gel pre-stained with GelRed^®^ Nucleic Acid Gel Stain (Biotium, CA, USA).

### 2.14. Establishment of DDX23-Knockout PK-15 Cell Line Using the Clustered Regularly Interspaced Short Palindromic Repeats ( CRISPR)/Cas9 System

The pSpCas9(BB)-2A-Puro (PX459) V2.0 plasmid expressing Cas9 (Addgene, plasmid 62988, Watertown, MA, USA) was digested with BbsI (NEB) and ligated to an annealed single guide RNA (sgRNA) oligonucleotide targeting porcine DDX23, as previously described [48]. The sgRNA sequence used in this study was designed with the online CRISPR design tool (http://crispr.tefor.net/), and the sequence was 5′-CACCGTAGGAAACGGCATCGGTCAA-3′. PK-15 cells were cultured in six-well plates at a density of 3 × 10^5^ cells/well, and the monolayer cells were transfected with the constructed plasmid (4 µg per well). After 48 h, the transfected cells were treated with 1.3 µg mL^−1^ puromycin, and the cells were passaged every 48 h with the administration of puromycin. The cells were counted, and 100, 200, and 300 cells were transferred to three 100 mm dishes and treated with puromycin. The single clones were picked with a cloning cylinder (Pyrex, San Nicolas de Los Garza, Nuevo Leon, Mexico) and transferred to a 48-well plate. When it grows 100 percent, they were passaged to 24 well plates; after every 48 h, cells were passaged subsequently to 12, 6 well, and finally to 25 mm plates. The cells were then stored in liquid nitrogen. The genomic DNA was extracted from the knockout cells with the Universal MiniBEST Genomic DNA Extraction Kit (TaKaRa). Primers were prepared based on the genomic DNA sequence of DDX23 (accession no. NC_010447): forward 5′-TAACACCCATGTGTCCTTAGGC-3′ and reverse 5′-TATTCCGATCTCGCTCCTTATC-3′. The genomic DNA of the cells cultured from a single clone was amplified with these primers. Eleven clone-derived samples were sequenced to ensure that the deletion was established in the cell line. The Western blot analysis was performed to confirm that DDX23 was not expressed in the DDX23-knockout cell line. Wild-type (WT) PK-15 cells were used as the control.

### 2.15. Statistical Analysis

Sequence data were analyzed with EditSeq, SeqMan, and MegAlign (DNA Star, Madison, WI, USA). Fold gene expression changes were calculated with the 2^−ΔΔCt^ method in Microsoft Excel 2019 (Redmond, WA, USA). The data presented in this paper are expressed as the means ± standard deviations (SD) of at least three replicates and were evaluated with two-tailed Student’s *t*-test in GraphPad PRISM software version 7 (La Jolla, CA, USA). Significant differences are presented as * *p* < 0.05, ** *p* < 0.01, or *** *p* < 0.001; “ns” indicates a nonsignificant difference (*p* > 0.05).

## 3. Results

### 3.1. DDX23 Interacts with the FMDV IRES

**Figure 1:**
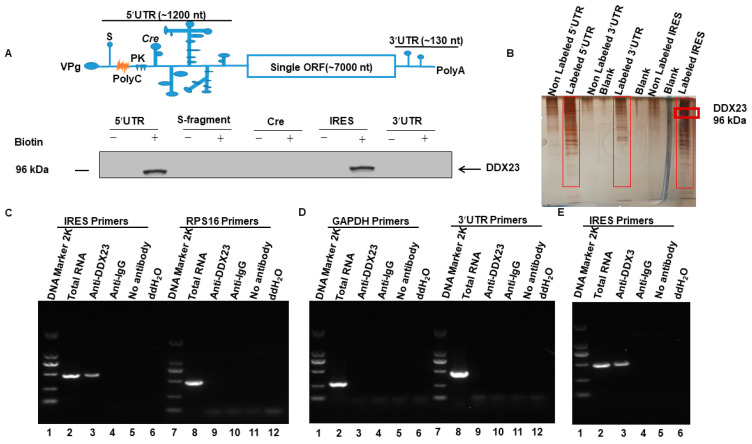
DEAD-box helicase 23 (DDX23) interacts with foot-and-mouth disease virus internal ribosome entry sites (FMDV IRES) and 5′(untranslated region) UTR. (**A**) Top, symbolic image of the FMDV genome, orange indicate PolyC. Bottom, cell lysates incubated separately with the biotin-labeled 5′UTR, S-fragment, cis replication element (cre), IRES, and 3′UTR of FMDV. Nonbiotinylated RNA probes were used as controls. After being pulled-down by streptavidin beads, the protein complex was dissolved for Western blotting. DDX23 was successfully pull-down with the FMDV 5′UTR and IRES. (**B**) Following pull-down assay, the eluted samples were run on SDS Page whereas the red rectangle indicates the size of the DDX23. The gel was silver-stained, and the bands were cut down and analyzed by Mass spectrometry. (**C**) PK-15 cells were infected with FMDV for 5 h. The cell lysate was subjected to immunoprecipitation assay with DDX23 or anti-IgG antibodies, no antibody, and ddH_2_O. RNA was extracted and subjected to RT-PCR using primers that were specific to IRES and RPS16. (**D**) PK-15 cells were infected with FMDV for 5 h. The cell lysate was subjected to immunoprecipitation assay with DDX23 or anti-IgG antibodies, no antibody, and ddH_2_O. RNA was extracted and subjected to RT-PCR using primers that were specific to *GAPDH* and 3′UTR. (**E**) PK-15 cells were infected with FMDV for 5 h. The cell lysate was subjected to immunoprecipitation assay with DDX3 or anti-IgG antibodies, no antibody, and ddH_2_O. RNA was extracted and subjected to RT-PCR using primers that were specific to IRES.

The host DEAD-box proteins are a recent point of focus for researchers because they bind to RNAs and alter different RNA–virus activities. The schematic diagram of the FMDV genome is shown in Figure 1A. To understand the direct interaction between DDX23 and the FMDV IRES, 5′UTR, and 3′UTR, we performed an RNA–protein pull-down assay. The final products were run on SDS gel and silver staining was performed (Figure 1B). The bands on the silver-stained gels were excised and analyzed with mass spectrometry. Several proteins interacted with the 5′UTR, IRES, and 3′UTR, which are shown in Tables S1–S3 in Supplemental materials. These results confirmed that DDX23 interacts with the FMDV 5′UTR and IRES, but not with its 3′UTR. Furthermore, the association of DDX23 with the IRES of FMDV was determined through RNA immunoprecipitation. PK-15 cells were infected for 5 h with FMDV at MOI of 0.5. The cellular lysate was prepared, and immunoprecipitation was performed using DDX23 or DDX3 antibodies. RNA was extracted from these immunoprecipitated samples and incorporated into RT-PCR. IRES, 3′UTR, and mRNA for RPS16, glyceraldehyde-3-phosphate dehydrogenase (*GAPDH*) were used as control. The FMDV IRES was identified in the samples immunoprecipitated with DDX23 or DDX3 antibodies (Figure 1C, lane 3, and Figure 1E lane 3). While RT-PCR did not detect RPS16, *GAPDH*, and 3′UTR in immunoprecipitated samples (Figure 1C, lane 9, Figure 1D lane 3, and 9 respectively), the IRES, RPS16, *GAPDH* and 3′UTR were not detected in anti-IgG, no antibody, and ddH_2_O (Figure 1C, lane 4, 5, 6, 10, 11, and 12, Figure 1D, lane 4, 5, 6, 10, 11, and 12, Figure 1E lane, 4, 5, and 6). These results confirm that DDX23 binds to the FMDV in infected cells.

### 3.2. DDX23 Interact with the Specific Regions of the FMDV IRES

**Figure 2:**
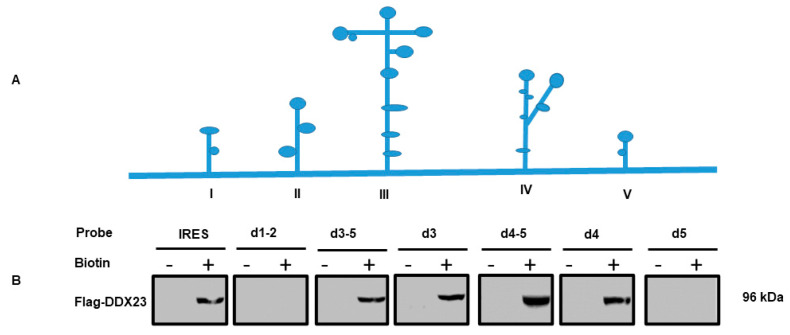
The regions of the FMDV IRES that interact with DDX23. (**A**) schematic diagram of FDMV IRES where different domains are shown. (**B**) Map of the DDX23 interaction region in the FDMV IRES. PK-15 cells were cultured on a 100 mm plate; at 70% confluence, the cells were transfected with Flag-DDX23, and 24 h post-transfection, the cell lysate was collected using RIPA buffer. The lysate was mixed with RNA probes spanning different regions of the FMDV IRES using streptavidin magnetic beads. Western blot assay was used with an anti-Flag antibody.

To illustrate the interaction between the specific regions of the FDMV IRES and DDX23, the regions of interaction with DDX23 in the FMDV IRES were mapped. Bearing in mind the FMDV IRES secondary structures as predicted by M-FOLD (Figure 2A), in vitro transcription technique was used to construct the truncated forms of FMDV IRES and labeling was achieved with biotin. Consequently, to analyze the interaction between various IRES truncated constructs and DDX23, the pull-down assay was performed. The pull-down results with biotinylated IRES truncated constructs showed that domain (s) d3-5, d3, d4-5, d4 interacted with the DDX23 but not domain 1–2 and d5 (Figure 2B). Suggesting that DDX23 interacts with domains III and IV of the FMDV IRES.

### 3.3. FMDV Infection Reduces DDX23 Protein Expression

**Figure 3:**
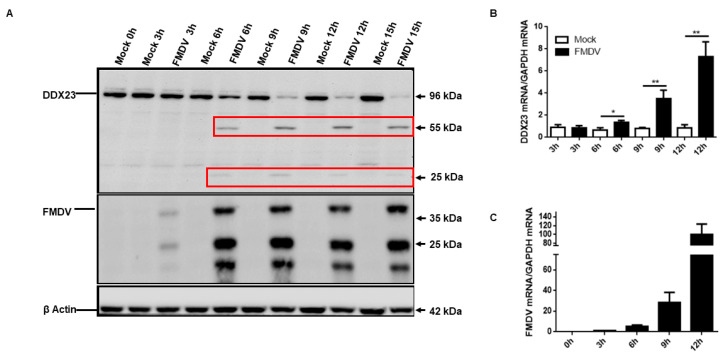
FMDV infection reduces DDX23 protein expression. (**A**) The PK-15 cells were cultured on 12 well plates following the monolayer growth. Cells were infected with FMDV at a multiplicity of infection (MOI) of 0.5. The samples were collected at the indicated time point, and Western blotting was performed. The red rectangles show the cleaved parts (55 and 25 kDa) of the DDX23. (**B**,**C**) RNA was extracted from the FMDV infected and mock-infected cells, and qRT-PCR was performed. DDX23 mRNA and FMDV mRNA levels were determined. Data represent the mean ± SD from three independent experiments (* *p* < 0.05, ** *p* < 0.01).

The interplay between FMDV and DDX23 had not been investigated, so we examined the dynamics of DDX23 during FMDV infection. PK-15 cells were infected with FMDV serotype O strain O/BY/CHA/2010 (GenBank accession number JN998085.1) at the multiplicity of infection (MOI) of 0.5, and the expression of DDX23 protein was measured. FMDV infection reduced the DDX23 protein level, and waning of DDX23 protein expression was observed at 6, 9, 12, and 15 hpi (Figure 3A). The cleaved part of DDX23 was observed at 6, 9, 12, and 15 hpi with approximately 55 kDa. We also measured the DDX23 mRNA levels during infection, which increased as infection progressed (Figure 3B), although this did not occur in the mock-infected cells. The interplay between FMDV and DDX23 mRNA was also evaluated, and as the level of FMDV mRNA increased; an increase in DDX23 mRNA was observed (Figure 3C). Our findings suggest that as the FMDV infection advances, it induces an increase in DDX23 mRNA. However, it significantly reduces DDX23 protein expression.

### 3.4. DDX23 Inhibits FMDV Replication

**Figure 4:**
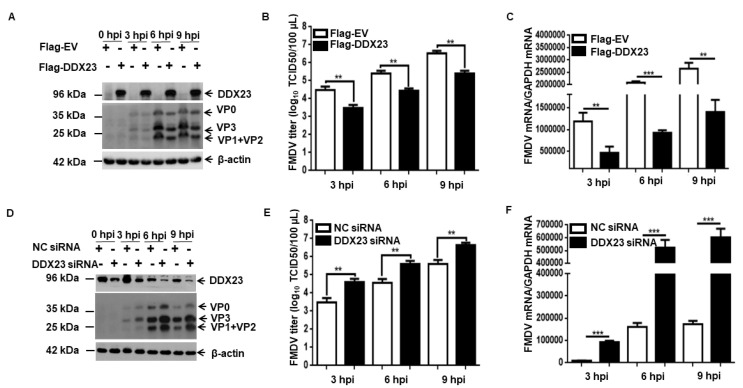
DDX23 inhibit FMDV replication. PK-15 cells were cultured on a 6-well plate and transfected with 3µg of Flag-DDX23 plasmid or Flag-empty vector (EV) as a control. (**A**) The cells were infected with FMDV at MOI of 0.5, samples were collected at 0, 3, 6, and 9 h, FMDV viral protein expression was determined by Western blotting. (**B**) DDX23 overexpressed and control PK-15 cells were infected with FMDV at MOI of 0.5. The supernatants were collected at indicated times, and virus yields were analyzed by tissue culture infection dose 50 (TCID_50_) assay. (**C**) viral mRNA was extracted from the PK-15 transfected cell and subjected to qRT-PCR. (**D**) PK-15 cells were transfected with DDX23 siRNA after 38h cells were infected with FMDV, at MOI of 0.5. The samples were collected at the indicated time point, and Western blotting was performed. (**E**) PK-15 cells were transfected with DDX23 small interfering (si)RNA or negative control (NC) siRNA (40 pmol). After FMDV infection, the supernatants were collected, and TCID_50_ was performed to determine the FMDV titer. (**F**) PK-15 cells were transfected with DDX23 siRNA or NC siRNA (40 pmol), and the FMDV mRNA level was determined by qPCR. The data presented in panels A and D is one of the triplicate experiments. Whereas the data in panel B, C, E, and F were repeated three times (** *p* < 0.01, or *** *p* < 0.001).

The marked reduction in DDX23 protein and the increase in DDX23 transcripts during FMDV infection suggest that DDX23 plays a vital role in FMDV infection. Therefore, we evaluated the mRNA levels, protein expression, and viral RNA titers of FMDV when DDX23 expression was upregulated. PK-15 cells were grown for 12 h and transfected with the DDX23-Flag-encoding plasmid with Lipofectamine LTX Plus Reagent (Invitrogen), according to the manufacturer’s protocol. After 24 h, the cells were infected with FMDV type O at an MOI of 0.5. Samples were collected at 0, 3, 6, and 9 hpi. The upregulation of DDX23 significantly reduced FMDV protein expression, viral titer and, mRNA level at each time point (Figure 4A–C). DDX23 knockdown was performed with DDX23 siRNA, using NC siRNA as a control. The cells were infected 35 h after knockdown with equal amounts of FMDV (MOI = 0.5), harvested at 0, 3, 6, and 9 hpi, and then subjected to Western blotting or qPCR analyses. It was evident that the FMDV proteins, viral titers, and mRNA level increased significantly (Figure 4D–F). Taken together, our findings demonstrate the importance of DDX23 in FMDV infection. Therefore, FMDV reduces the DDX23 level to enhance its replication.

**Figure 5:**
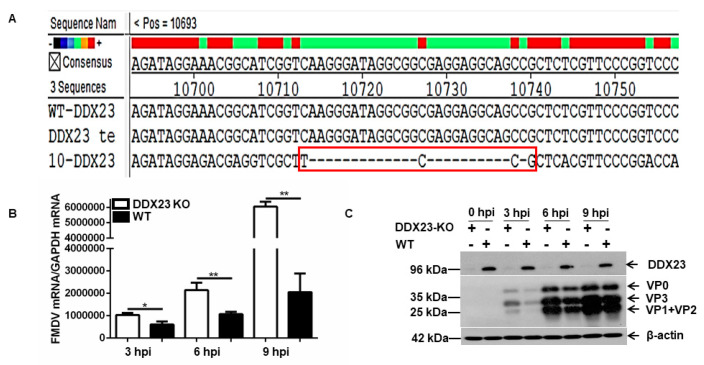
DDX23-knockout (KO) cells show a significant increase in FMDV mRNA and protein level. PK-15 cell line was used to establish a DDX23 knockout stable cell line. (**A**) DDX23 stable cell line was sequenced using Sanger sequencing technology. It was aligned with the DDX23 genome sequence from NCBI and DDX23 wild type (WT), the red rectangle indicates that 25 nucleotides were successfully deleted from the sequence. (**B**) The established DDX23-KO and WT cells were grown on a six-well plate and grown for 30 h and infected with FMDV at MOI of 0.5, and the samples were collected at 0, 3, 6, and 9 h, mRNA was collected, and qPCR was performed. (**C**) The established DDX23-KO and WT cells were grown on a six-well plate and grown for 30 h and infected with FMDV at MOI of 0.5, and the samples were collected at 0, 3, 6, and 9 h, and Western blot was performed. These experiments were repeated at least three times (* *p* < 0.05, ** *p* < 0.01).

A DDX23-knockout (DDX23-KO) PK-15 cell line was established by manipulating CRISPR Cas9 system to determine the inhibitory effect of DDX23 on FMDV replication. DNA sequencing and Western blotting were used to confirm the successful knockout of DDX23. The sequencing results showed that 25 nucleotides were deleted from the first exon of DDX23 (Figure 5A). The DDX23-KO and WT PK-15 cells were infected with FMDV (MOI = 0.5), and the expression of FMDV mRNAs and protein were determined. FMDV mRNAs and proteins were significantly more highly expressed in the DDX23-KO cells than in the WT PK-15 cells (Figure 5B,C). These data demonstrate the antiviral effects of DDX23 during FMDV infection.

### 3.5. DDX23 Suppresses IRES-Dependent Translation and Viral Replication

**Figure 6:**
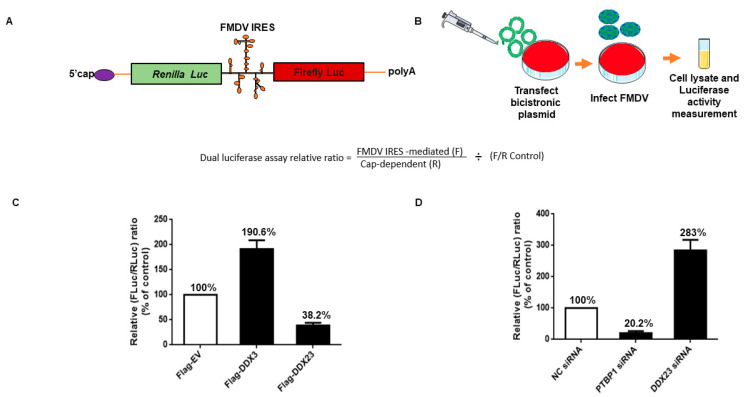
DDX23 suppresses FMDV cap-independent translation. (**A**) Schematic representation of dual-luciferase Bicistronic reporter plasmid. (**B**) schematic representation of dual luciferase assay. (**C**) PK-15 cells were grown on 24 well plates after 12 h, the cells were co-transfected with Flag-DDX23 plasmid (0.5 µg) and bicistronic FMDV IRES plasmid (0.5 µg) or Flag-DDX3 (0.5 µg) as a positive control and Flag-EV (0.5 µg) as a negative control. The cell lysates were collected and subjected to luciferase activity measurement. (**D**) PK-15 cells were cultured on 24 well plates when the cells reached 70% confluency, DDX23 siRNA (40 pmol) was used to knockdown, and similarly, siRNA polypyrimidine tract-binding protein 1 (PTBP1) was used as control positive, and NC siRNA was used as a negative control. Cells were lysed with passive lysis buffer, and the samples were analyzed through Dual-Glo^®^ Luciferase Assay System (Promega). The data presented above are from three independent experiments.

To investigate whether DDX23 is involved in FMDV IRES-directed translation, a bicistronic luciferase plasmid (psiCHECK-FMDV) containing a cap-dependent *Renilla* luciferase (RLuc) gene and an FMDV IRES-dependent firefly luciferase (FLuc) gene was constructed (Figure 6A). The relative FMDV IRES activity was detected as the ratio of FLuc expression to RLuc expression. PK-15 cells were co-transfected with a Flag-DDX23-expressing plasmid and bicistronic plasmid. The lysates were incorporated into luciferase activity measurement (Figure 6B). A Flag-DDX3-expressing plasmid was used as a positive control, and its overexpression increased the activity up to 190.6%. It is reported that DDX3 upregulates FMDV IRES activity [46]. We observed a significant reduction in FMDV IRES activity recorded at 38.2% when DDX23 was overexpressed (Figure 6C). However, when DDX23 was knocked down, a significant increase of up to 283% was recorded in IRES activity. PTBP1 was used as the positive control, and its knockdown significantly reduced the FMDV IRES activity up to 20.3%. PTBP is reported to upregulate the activity of IRES [14,49] (Figure 6D). In summary, our results show that DDX23 suppresses the FMDV IRES-dependent translation.

### 3.6. DDX23 Translocates during FMDV Infection

**Figure 7:**
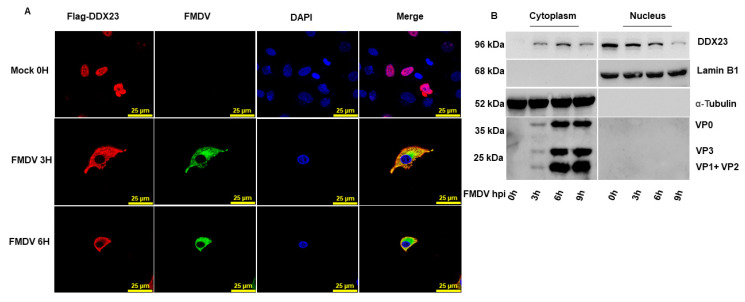
DDX23 shows interaction with FMDV. (**A**) PK-15 cells were cultured on glass-bottom cell culture dishes. Cells were transfected with Flag DDX23 (2 µg) at 50–60% confluency 24 h post-transfection and then infected with FMDV at MOI of 5. The specimens were fixed with 4% paraformaldehyde at 0, 3, and 6 h and the cells were double immunostained for Flag-DDX23 (red), FMDV (green), and nuclei were stained blue, whereas pink color indicate DDX23 and nuclei merged and yellow indicate DDX23 and FMDV merged. (**B**) PK-15 cells were grown up to a monolayer on 100 mm plates and infected with FMDV at MOI of 0.5. The cells were harvested at 0, 3, 6, and 9 h, and the nuclear cytosol fractionation assay was performed.

Previous studies have shown that helicase translocation is an important mechanism in virus-host interaction. The translocation of proteins to the cytoplasm is considered to be linked with the replication of the virus. Therefore, we tried to investigate the association between DDX23 and FMDV. PK-15 cells were transfected with DDX23-Flag-encoding plasmid. After 24 h, the cells were infected with FMDV (MOI = 5). The cells were fixed with 4% paraformaldehyde and subjected to an immunofluorescence antibody test. DDX23 had dramatically translocated from its original nuclear location to the FMDV-infected cells’ cytoplasm, where it interacts with viral RNA. In contrast, in mock-infected cells, DDX23 was only detected in nuclei (Figure 7A). Furthermore, the cytoplasmic translocation of DDX23 was confirmed through nuclear cytosol fractionation assay. Cells were cultured on 100 mm cell culture dishes until they reached a monolayer. The cells were infected with FMDV at an MOI of 0.5. The cell lysates were collected at the indicated time interval. DDX23 translocate to the cytoplasm with FMDV infection; however, due to the degradation by FMDV, its expression reduced with increasing time of infection, as shown in Figure 7B. These results demonstrated the relationship between DDX23 and FMDV, which affects the virus.

### 3.7. FMDV 3C^pro^ Interacts with DDX23, and Its Proteinase Activity Reduced DDX23 Expression

**Figure 8:**
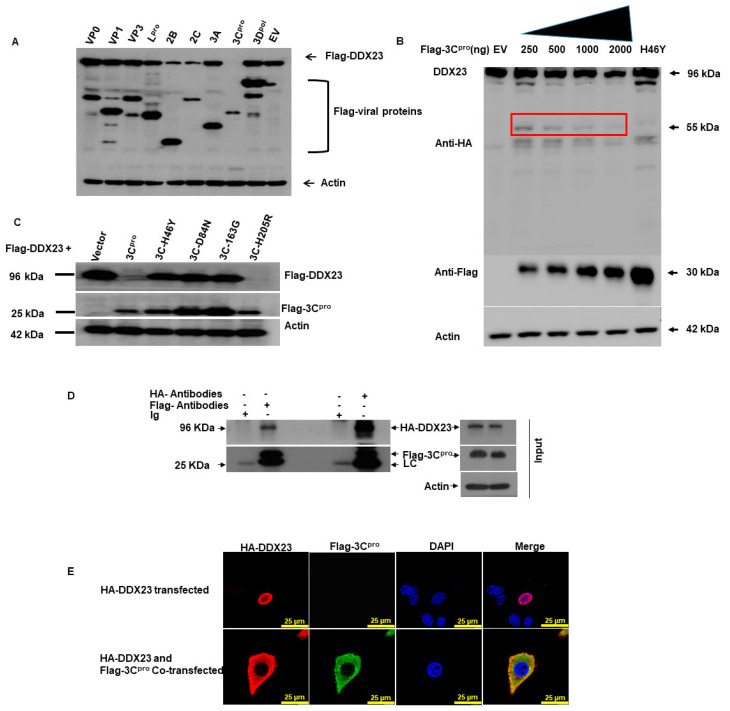
FMDV 3C proteinase (3C^pro^) degrades DDX23, and 3C proteinase enzymatic activity is responsible for this degradation, whereas 3C^pro^ interacts with DDX23. (**A**) PK-15 cells were cultured on six-well plates after reaching 70% confluence. The cells were co-transfected with Flag-DDX23 (2 µg), along with FMDV protein plasmids (VP0, VP1, VP3, L^pro^, 2B, 2C, 3A, 3C^pro^, 3D^pol^) or Flag-EV (2 µg). Samples were collected after 36 hpt and analyzed through Western blotting. (**B**) PK-15 cells were cultured on a six-well plate at 70% confluence. The cells were co-transfected with hemagglutinin (HA)-DDX23 (2 µg), Flag-3C^pro^ with increasing concentration (250 ng, 500 ng, 1000 ng, and 2000 ng) Flag-EV, and H46Y. Samples were collected after 24 hpt and resolved on SDS-PAGE and used in Western blot assay with an anti-HA and or Flag antibodies. the red rectangle shows the cleaved part of the DDX23. (**C**) PK-15 cells were Co-transfected with Flag-DDX23 and FMDV Flag-3C, 3C-H46Y, 3C-D84N, 3C-163G, 3C-H205R or Flag-EV. The expression of proteins was detected 36 hpt by Western blotting. (**D**) PK-15 cells were grown on 10-cm dishes; cells were Co-transfected with HA-DDX23 expressing plasmid 8 µg and Flag 3C expressing plasmid 5 µg. After 24 h, the cells were harvested, and the lysates were immunoprecipitated with anti-HA, anti-Flag antibodies, and anti-Ig. The complexes were analyzed by Western blotting using anti-HA, anti-Flag, and Ig antibodies. (**E**) PK-15 cells were cultured on a glass-bottom cell culture dish when it reached the confluency of 50–60%. The cells were co-transfected with HA-DDX23 (1.5 µg) and Flag-3C (1.5 µg). The expression of HA-DDX23 and Flag-3C was detected by Immunofluorescent assay (IFA) analysis at 30 hpt. Cells were double immunostained for HA-DDX23 (red), Flag-3C (green), and nuclei were stained (blue), whereas pink color indicate DDX23 and nuclei and yellow indicate DDX23 and 3C^pro^. These experiments were repeated at least three times.

DDX23 protein expression is reduced by FMDV infection. Therefore, we screened for the FMDV protein(s) that cause this reduction in DDX23. PK-15 cells were co-transfected with plasmid encoding FMDV-VP0, VP1, VP3, L, 2B, 2C, 3A, 3C, 3D, or Flag-EV and the plasmid encoding Flag-DDX23. The cells were harvested after 24 h and analyzed with Western blotting. Of the FMDV proteins, 3C^pro^ significantly reduced the level of Flag-DDX23 (Figure 8A). These results show that the FMDV 3C protein reduces DDX23, thus antagonizing the host’s antiviral effect.

3C^pro^ with increasing concentration of 250 ng, 500 ng, 1000 ng, and 2000 ng were co-transfected with hemagglutinin (HA)-DDX23 plasmid in PK-15 cells, 24 hpt the cells were harvested and analyzed through Western blotting. The waning of DDX23 protein expression was observed at each concentration of 3C^pro^ (Supplementary File; Figure S1). Approximately 55 kDa cleaved part was observed. However, such cleavage part was not observed in the catalytically inactive 3C^pro^ mutant (H46Y) and EV samples (Figure 8B).

The 3C^pro^-dependent reduction in DDX23 and the involvement of the enzymatic activity of 3C^pro^ were investigated further. Plasmids encoding various 3C^pro^ mutants, with (H205R) and without (H46Y, D84N, H163R) enzymatic activity, were constructed. The 3C^pro^ mutants without enzymatic activity (H46Y, D84N, H163R) did not affect DDX23 expression. In contrast, the 3C^pro^ WT and the 3C^pro^ mutant having enzyme activity (H205R) abolished DDX23 protein expression (Figure 8C), suggesting that the 3C^pro^-dependent reduction in DDX23 is strongly dependent on the enzyme activity of 3C^pro^.

FMDV 3C^pro^ reduces DDX23 protein expression; therefore, we investigated the interaction between DDX23 and 3C^pro^. PK-15 cells were co-transfected with plasmids encoding HA-DDX23 and Flag-3C^pro^. After 24 h, the cell lysates were immunoprecipitated with anti-Flag and anti-HA antibodies for Western blotting analysis. DDX23 interacted with FMDV 3C^pro^ (Figure 8D). Forward immunoprecipitation was performed with an anti-Flag antibody, which pulled down the HA-DDX23 protein, whereas reverse immunoprecipitation reaction was performed with an anti-HA antibody that successfully pulled down the Flag-3C^pro^. These results suggest a strong correlation between DDX23 and FMDV 3C proteinase. We also confirmed this interaction with confocal microscopy. For this purpose, PK-15 cells were cultured in a glass-bottomed cell culture dish and co-transfected with plasmids encoding HA-DDX23 and Flag-3C^pro^ or only HA-DDX23. An Immunofluorescent assay was performed; cells were incubated overnight at 4 °C with anti-Flag and anti-HA primary antibodies. The specimens were next stained with fluorochrome-conjugated secondary antibodies at 37 °C for 1 h. The results showed that red-stained DDX23 interacted with green-stained FMDV 3C^pro^. However, the cells transfected only with HA-DDX23 showed no change in the position of DDX23 residing in the nucleus (Figure 8E).

### 3.8. Involvement of the Lysosomal Pathway in FMDV-Dependent Reduction of DDX23

**Figure 9:**
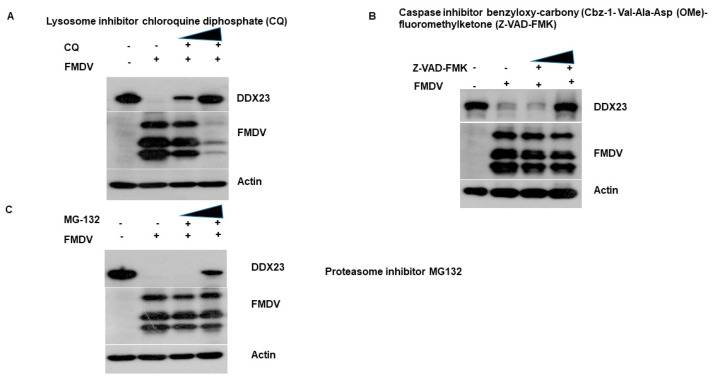
FMDV induces DDX23 reduction through the lysosomal pathway. (**A**–**C**) PK-15 cells were cultured on six-well plates. Cells were mock or FMDV infected on reaching to a monolayer at MOI of 0.5 and were treated in the presence or absence of the lysosome inhibitor CQ (50 or 100 µM), the caspase inhibitor, caspase inhibitor benzyloxy-carbonyl (Cbz-1-Val-Ala-Asp)-(OMe)-fluoromethylketone (Z-VAD-FMK) (10 or 50 µM) or proteasome inhibitor MG132 (2 or 20 µM) for 12 h. the expression of endogenous DDX23 and viral proteins were detected by Western blotting.

To assess the involvement of the lysosomal, proteasomal, and caspase pathways in the FMDV-dependent reduction of DDX23, the lysosome inhibitor chloroquine diphosphate (CQ), the proteasome inhibitor MG-132, and the caspase inhibitor, caspase inhibitor benzyloxy-carbonyl (Cbz-1-Val-Ala-Asp- (OMe)-fluoromethylketone (Z-VAD-FMK) was used. PK-15 cells were infected with FMDV at MOI of 0.5, and the inhibitors were added after one h. The cells were harvested at 12 hpi for Western blotting analysis. The addition of the lysosomal inhibitor restored DDX23 protein expression in the cells. In contrast, the lower concentrations of caspase and proteasome inhibitors did not restore DDX23. However, their high concentrations did restore protein expression (Figure 9A–C). The interesting finding with the lysosomal inhibitor was that as FMDV protein expression decreased, DDX23 protein expression was correspondingly restored, which supports the antiviral effect of DDX23. These results suggest that FMDV potentially uses the lysosomal pathway to degrade DDX23 protein to promote its replication.

## 4. Discussion

The critical feature in *Picornaviruses* infection is IRES-mediated translation regulation, which critically impacts viral pathogenicity, virulence, and tissue tropism [50]. However, the mechanism of IRES-mediated translation is poorly understood. In the present study, we identified DDX23 as a novel ITAF for the *Picornavirus* FMDV, and its interaction with FMDV IRES was evaluated for the first time. Furthermore, the proteomic data showed that a large number of proteins were associated with the IRES of FMDV. On the other hand, 3C^pro^ degraded DDX23 to antagonize its antiviral activity.

DDX23 plays diverse roles in cellular processes, including genome stability, RNA translation, and splicing via binding with RNA and regulation of transcription [25,51], as well as suppression or promotion of virus replication [31]. Dengue virus is a single positive-stranded RNA virus, and DDX23 negatively modulates its replication. An earlier study suggested that the non-structural protein NS5 of Dengue virus interacts with the core components (DDX23 and CD2BP2) of the U5 snRNP particle, and manipulates its alternative splicing activity by changing the inclusion/exclusion ratio [28]. In contrast, the RNA-interference-mediated suppression of DDX23 significantly reduced Astrovirus genomic and agenomic RNA levels, the synthesis of the structural protein VP90, and the viral yield [33]. Therefore, we were very curious to know the association between DDX23 and FMDV. We observed that the knockdown of DDX23 significantly increased FMDV replication, whereas its overexpression suppressed FMDV RNA synthesis and protein expression. The difference in the regulation of both viruses might be due to the different virus and cell conditions. Here, we reported the mechanism by which DDX23 affects FMDV replication, confirmed with mass spectrometry and RNA pull-down assay, which demonstrated that the FMDV IRES interacts with DDX23. Further investigation confirmed that DDX23 interacted with the FMDV IRES domain III and IV. Meanwhile, we also observed a novel function of DDX23, in which overexpression reduced FMDV IRES driven translation through bicistronic dual-luciferase assay, while knockdown increased. IRES facilitates the viral RNA translation initiation via a cap-independent mechanism. These IRES’s require different cellular proteins, known as ITAFs, and they can acquire various secondary structures to help virus replication [52,53]. Each part of the IRES has a different function, for example, FMDV domain I is mainly involved in the replication of the virus, whereas in domain II, the pyrimidine tract sequence function is pyrimidine binding protein recruitment [6,54]. However, domain III of the FMDV IRES is crucial for the attachment with IRES transacting factors and RNA-RNA interaction, facilitated by its long and flexible structure. Hence, this flexibility is utilized to compel various ITAFs to bind and efficiently modulates IRES activity [55].

The DEAD-box helicase DDX23 function is RNA splicing. Therefore, it localizes to the nuclei of cells [30]. Previously, it was considered that the cytoplasmic re-localization of proteins is due to the inhibition of cellular transcription induced by viruses [56]. However, when actinomycin D was used to inhibit the cellular transcription, the re-localization was even more dramatic in infected cells than treated with actinomycin D, confirmed that cellular transcription inhibition is not the sole player in the cellular re-localization of proteins [57]. DDX23 is a double-stranded RNA sensor, so treatment with poly(I:C) or vesicular stomatitis virus (VSV) causes the helicase to translocate to the cytoplasm of the cell [47]. Previously it was shown that the nuclear protein splicing factor proline and glutamine rich (SFPQ) does not translocate to the cytoplasm when cells were stimulated with polyinosinic: polycytidylic acid (polyI:C). However, infection with encephalomyocarditis virus causes translocation of SFPQ into the cell cytoplasm [58]. On the other hand, non-shuttling proteins have also shown this movement, e.g., hnRNP C, which re-localized to the cytoplasm with poliovirus infection. However, the kinetics is slower than that of the shuttling proteins [57]. Herein, we investigated the nucleo-cytoplasmic translocation of DDX23 in FMDV infected cells, and detected the translocation of the DDX23 to the cytoplasm of cells infected with FDMV. The cytoplasmic translocation of DDX23 and subsequent reduction in its protein expression may enhance cellular apoptosis, benefiting viral replication. Taken together, it suggests thatDDX23 is not only involved in RNA splicing in the nucleus but translocate to the cytoplasm to affect the viral replication at the posttranscriptional level.

As RNA viruses enter the cells′ cytoplasm, the RNA virus sensor RIG-I senses them and induces type I interferon response (*IFN1*). RIG-I is an essential molecule in the innate immune system [59,60]. A significant increase in the mRNA level of RIGI was observed when PK-15 cells were infected with FMDV. However, a dramatic reduction in RIG-1 protein expression was observed. FMDV proteins L^pro^, 2B, and 3C^pro^ significantly reduced RIG-I expression [61]. DDX23 is a viral RNA sensor and involved in innate immune response [30], therefore, we determined the DDX23 protein expression and its mRNA level. We showed that FMDV infection dramatically reduces DDX23 protein expression, whereas its mRNA expression was significantly enhanced. Furthermore, FMDV protein 3C^pro^ dramatically degraded DDX23 after being interacted with it, which suggest that the increased DDX23 mRNA antagonize FMDV infection through the cellular mechanism and possibly involved in the innate immune response. The FMDV viral 3C proteinase was found to be a potential target for antiviral drugs [43], which is encoded by a highly conserved gene in the viral genome [34]. The catalytically active sites in 3C^pro^, D84, C163, and H46Y process the FMDV polyproteins and host cellular proteins [37,38,39,40,41]. FMD 3C^pro^ dependent degradation of PKR and ATG5-ATG12 has already been reported, which confirms that this degradation is significant to subvert the antiviral activity of the aforementioned proteins, furthermore, the host proteins PKR and ATG 5 play an essential role in innate immunity, suggesting that 3C^pro^ battle on the front line against these antiviral proteins to antagonize its antiviral activity and help the virus replicate. [62,63].

The growing body of study has shown that host protein degradation is mainly dependent on several pathway/s. During FMDV infection, the protein kinase R (PKR) degradation occurs via the lysosomal pathway, independent of the enzymatic activity of 3C^pro^ [62]. In another study, the expression of host protein DDX1 was reduced in cells infected with FMDV [64]. However, the degradation of the host protein was independent of the proteasome, caspase, and, lysosomal pathways. In a previous study, it was shown that RIG-I degradation was independent of the aforementioned pathways [61]. Therefore, we investigated whether DDX23 degradation is associated with these pathways. We show that FMDV infection significantly reduced host DDX23 expression by degrading the protein via the lysosomal pathway. The different scenarios in both studies could be due to the different proteins to which viruses respond differently. Previously, it was shown that Influenza A virus infection induces the degradation of eIF4B however, the treatment of chloroquine diphosphate restored eIF4B protein expression and no decrease was observed in viral protein expression [65]. In contrast, in the present study, we found that the treatment of chloroquine diphosphate restored the DDX23 protein expression but significantly reduced the FMDV viral protein expression. Similarly, a previous study showed that FMDV infection reduced the RIG-I protein expression whereas chloroquine diphosphate, MG132 or Z-VAD-FMK, none of them restored the RIG-I protein expression while no decrease in the viral protein expression was observed [61]. The difference in both the studies might be due to different host proteins which may pose different strategies to encounter the virus. DDX23 is a multifunctional protein and the current study opens up the gateway for further research on the antiviral mechanism of DDX23.

In this study, we concluded that FMDV infection inhibited DDX23 expression. DDX23 is a novel FMDV-ITAF, which negatively regulated FMDV IRES translation and replication. Meanwhile, FMDV 3C^pro^ degraded the host protein DDX23, and antagonized its antiviral activity, whereas FMDV degraded DDX23 via the lysosomal pathway. Our findings suggested that the anti-FMDV activity of DDX23 could be utilized to develop potent antiviral therapy.

## Supplementary Materials

The following are available online at https://www.mdpi.com/1999-4915/12/12/1348/s1, Table S1: proteins interacting with the IRES of the FMDV. Table S2: proteins interacting with the 5′UTR of the FMDV. Table S3: proteins interacting with the 3′UTR of the FMDV. Figure S1: 3C^pro^ degrades DDX23 protein expression.
