# Supplementary material for: The DDX23 Negatively Regulates Translation and Replication of Foot-and-Mouth Disease Virus and Is Degraded by 3C Proteinase
Source: Viruses. 2020 Nov 25;12(12):1348. doi: 10.3390/v12121348 (PMC7760909; doi:10.3390/v12121348)

Table S1: The proteins shown are interacting with the IRES of FMDV

Protein Name	Seq. Coverage %age	Accession No.	Function
APEX1	6	XP_020953698.1	Single stranded RNA metabolism
ARHGEF2	4.1	NP_001121935.1	Innate immune response, cell cycle regulation
DDX17	33.9	XM_021091487	mRNA splicing, defense response to virus
DDX23	10.2	JAA74080.1	Pre-mRNA splicing
DHX29	0.5	XP_003133996.1	Translation initiation
EIF6	17.1	NP_001230506.1	Stimulatory translation initiation factor
Gag	19.5	AGK89510.1	Nucleic acid binding, viral process
GTPBP4	13.1	XP_003357838.1	Protein stabilization
HSPA1B	10.6	NP_998931.1	Protection of the proteome from stress, virus receptor activity
IQGAP1	3.4	XP_020954569.1	Cytoskeleton
MRTO4	7.9	PHH53476.1	rRNA processing
NOL6	2.4	XP_003130726.1	rRNA processing
PES1	1.7	NP_001231361.1	rRNA processing
RACK1	13.2	NP_999497.1	Translational repression, Inhibits cell growth
RCC1	4.9	XP_020951334.1	Cell division, viral process
RPL10A	29	BAA19211.1	Translation initiation
RPL15	4.7	EGJ70443.1	Translation initiation, RNA binding
RPL27	12.5	NP_001090948.1	Translational initiation, rRNA processing
RPLP0	17.3	NP_001092068.1	Translation initiation
RPS8	42.8	XP_003128108.1	translational initiation
RPS9	15.5	XP_020950538.1	Translation regulator activity
RRS1	4.1	XP_003125652.1	Ribosomal large subunit assembly
SARNP	9.5	JAG69200	cell cycle progression
SMARCA4	0.9	JAA53686.1	Transcriptional activation and repression
TOP2A	3.3	NP_999049.1	Positive regulation of apoptotic process
VAPB	4.9	NP_001116685.1	Cellular calcium ion homeostasis
WDR12	2.8	XP_013839854.1	rRNA processing
YBX1	12.3	AER27829.1	Transcription of numerous genes

Table S2: the proteins shown are interacting with 5’UTR of FMDV

**Protein Name**	**Seq. Coverage %age**	**Accession No.**	**Function**
ABCE1	3.7	XP_003129218.4	Defense response to virus translation initiation
ABCF1	12	NP_001116541.**1**	Translation initiation
ADAR	6.2	JAA53561.1	Positive regulation of viral genome regulation
CHERP	3.2	XP_020939241.1	mRNA splicing, calcium homeostasis
CPSF2	9.8	XP_020955286.1	Pre-mRNA 3'-end formation, mRNA splicing
CWC15	5.2	XP_003129801.1	Pre-mRNA splicing
DDX17	42.1	XM_021091487	mRNA Splicing, defense response to virus
DDX23	12.7	JAA74080.1	Pre-mRNA splicing
DDX41	3.9	AGK93040	Post-transcriptional gene expression
DDX6	14.1	JAA74191.1	mRNA degradation, viral RNA genome packaging
EIF3B	2	JAA74076.1	IRES-dependent viral translational initiation
EIF3D	8	NP_001231652.1	Translation initiation
ELAVL1	10.4	NP_001182263.1	mRNA stabilization, intracellular mRNA localization
FUBP3	5.1	AEI54345.1	Positive regulation of gene expression, transcription
MYO6	6.9	XP_020938322.1	Positive regulation of transcription RNA polymerase II
PPP1R10	2.4	NP_001116637.**1**	Protein phosphatase inhibitor, RNA binding
RAE1	4.3	NP_001098766.1	Binds mRNA, viral process, cell division
RBM10	3	JAG69227.1	mRNA splicing
RBM25	6.5	JAG69225.1	RNA Splicing
RPL117	9	NP_001001638.1	Translation initiation, rRNA processing
SRSF10	9.2	AEI98875.1	regulation of mRNA splicing
TRA2A	7.9	ABI96194.1	Pre-mRNA splicing
TRIM25	3.4	XP_005657028.3	Innate immune defense against viruses
VCP	5.1	NP_999445.1	Viral genome replication, autophagy

Table S3: the proteins shown are interacting with 3’UTR of FMDV

**Protein Name**	**Seq. Coverage %age**	**Accession No.**	**Function**
DDX18	1.1	XP_005654221.2	Translation initiation, spliceosome assembly
DDX21	9.2	JAA53623	Double-stranded RNA binding, defense response to Virus
DHX36	3.4	JAA74136.1	Defense response to virus
EIF2S3	8.1	XP_020936181.1	Translation initiation, RNA binding
HNRNPD	21.6	XP_020957542.1	mRNA splicing, mRNA transcription
HNRNPR	26.4	JAA74142	mRNA splicing, mRNA processing
HSPA6	8.1	NP_001116599.1	Cellular response to heat
NONO	7	JAA53753	mRNA processing, RNA splicing
PABPC1	30.7	XP_001927782.1	Pre-mRNA splicing, Translation initiation
PABPC4	17.4	XP_005665309.1	Translation, mRNA 3’UTR binding
PRPF19	5	ACB59178.1	mRNA splicing
PTBP1	19.2	XP_005661470.**1**	Pre-mRNA splicing, IRES dependent viral Translational initiation
RBBP4	10.4	XP_013854580.1	Regulation of cell cycle
RPL18	7.8	ABD77175.1	Cytoplasmic translation
RPL7	8.3	AAS55898.1	Translation initiation, rRNA processing
VIM	42.7	JAG69056.1	Positive regulation of translation, viral process

Figure S1: 3C^{pro} degrades DDX23 protein expression

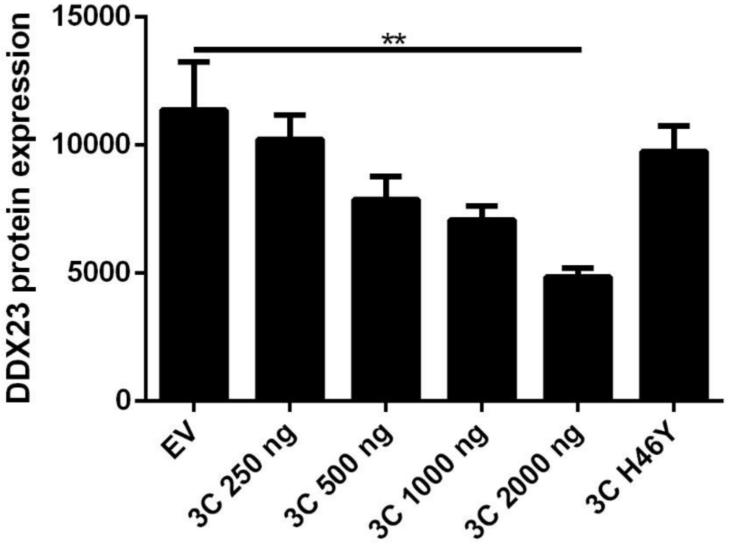

Supplement: Supplementary file 1 [file viruses-12-01348-s001.pdf]
